# Calling differentially methylated regions from whole genome bisulphite sequencing with DMRcate

**DOI:** 10.1093/nar/gkab637

**Published:** 2021-07-28

**Authors:** Timothy J Peters, Michael J Buckley, Yunshun Chen, Gordon K Smyth, Christopher C Goodnow, Susan J Clark

**Affiliations:** The Garvan Institute of Medical Research, 384 Victoria St, Darlinghurst, NSW 2010, Australia; UNSW Sydney, Sydney 2052, Australia; The Garvan Institute of Medical Research, 384 Victoria St, Darlinghurst, NSW 2010, Australia; UNSW Sydney, Sydney 2052, Australia; The Walter and Eliza Hall Institute of Medical Research, Parkville, VIC 3052, Australia; Department of Medical Biology, The University of Melbourne, Melbourne, VIC 3010, Australia; The Walter and Eliza Hall Institute of Medical Research, Parkville, VIC 3052, Australia; School of Mathematics and Statistics, The University of Melbourne, Melbourne, VIC 3010, Australia; The Garvan Institute of Medical Research, 384 Victoria St, Darlinghurst, NSW 2010, Australia; School of Medical Sciences and Cellular Genomics Futures Institute, UNSW Sydney, NSW 2052, Australia; The Garvan Institute of Medical Research, 384 Victoria St, Darlinghurst, NSW 2010, Australia; St. Vincent’s Clinical School, Faculty of Medicine, UNSW Sydney, NSW 2010, Australia

## Abstract

Whole genome bisulphite sequencing (WGBS) permits the genome-wide study of single molecule methylation patterns. One of the key goals of mammalian cell-type identity studies, in both normal differentiation and disease, is to locate differential methylation patterns across the genome. We discuss the most desirable characteristics for DML (differentially methylated locus) and DMR (differentially methylated region) detection tools in a genome-wide context and choose a set of statistical methods that fully or partially satisfy these considerations to compare for benchmarking. Our data simulation strategy is both biologically informed—employing distribution parameters derived from large-scale consortium datasets—and thorough. We report DML detection ability with respect to coverage, group methylation difference, sample size, variability and covariate size, both marginally and jointly, and exhaustively with respect to parameter combination. We also benchmark these methods on FDR control and computational time. We use this result to backend and introduce an expanded version of DMRcate: an existing DMR detection tool for microarray data that we have extended to now call DMRs from WGBS data. We compare DMRcate to a set of alternative DMR callers using a similarly realistic simulation strategy. We find DMRcate and RADmeth are the best predictors of DMRs, and conclusively find DMRcate the fastest.

## INTRODUCTION

DNA methylation is one of the first characterized epigenetic control modifications in eukaryotic organisms (1,2), and the investigation of this process is a central part of current biological and medical research (3–5). Single molecule DNA methylation profiles can be obtained via clonal sequencing of bisuphite treated DNA (6,7) or whole genome bisulphite sequencing (WGBS) (8,9). Unmethylated cytosines are converted to uracils in bisulphite treated DNA and subsequently to thymines after PCR amplification, whereas methylated cytosines are resistant to bisulphite treatment and so remain as cytosine residues. Sequencing of bisulphite treated DNA has become a gold standard process in determination of DNA methylation status in experimental and clinical samples. In an experimental context, bisulphite-treated DNA sequence reads are aligned to a reference genome, and for each possible methylation site in that sample, two tallies are produced: a count of cytosines *C* indicating methylation at this site, and a count of thymines *T* indicating bisulphite conversion of cytosine, hence no methylation at this site. Eukaryotic DNA methylation occurs primarily at CpG sites, of which the human reference has approximately 28 million. Therefore, a typical unabridged whole genome bisulphite sequencing (WGBS) dataset, for an experiment with *n* human samples, consists of a *p* × 2*n* array of read counts, where *p* ≈ 2.8 × 10^7^. The *C* + *T* total at each CpG site for each sample is the total number of reads aligning to that CpG site and is termed the coverage. If *C* + *T* > 0, the ratio $\frac{C}{C+T}$ is the observed methylation fraction.

Biological hypotheses motivate the need for inferences to be derived from these data sets. The central phenomenon of interest is differential methylation (DM), the counterpart of, in gene expression experiments, differential expression (DE). Most, if not all, hypotheses in the DE space are applicable to DM. In simple experiments, DM is the difference in methylation fraction between two experimental conditions. More generally, DM is an association of methylation fraction with an experimental factor. This may be a pairing factor, such as when tumour methylation is compared to matched non-cancerous tissue amongst a patient population. Biological covariates such as age or sex may need to be added to the statistical model as a corrective measure, or a continuous response such as age or body mass index (BMI) may itself be the variable of interest. More complex experimental hypotheses, such as post-hoc contrasts between two phenotypes where variation is estimated jointly from three or more groups (10), and interaction effects between two variables (such as the effect of a drug over a time period, compared to a control group) may be required by the study. We propose that a statistical DM detection tool must be flexible enough to infer results from most, if not all these different types of hypotheses, and this informs our choice of tools for benchmarking for this study.

The spatial distribution of DM markers across the eukaryotic genome is not random amongst CpG sites. Rather, differentially methylated loci (DMLs) tend to clump together in groups, giving the effect (such as when viewed in a genome browser) of a contiguous differentially methylated region, or DMR (11). Certain domains of the reference sequence may be categorized into CpG islands and shores by density-based segmentation (12), but these domains do not constitute a precise functional unit like, for example, an exon. Transcriptional units such as exons are explicitly defined at nucleotide resolution by precise molecular properties. In the case of DMRs, no such delimiters exist, and hence they must be defined in addition to modelling the differential signal. One option may be pre-defining regions of interest of the genome to test for DM (13), but this introduces a selection bias and hence the results of these analyses are not controlled for false discovery rate (FDR) at a genome-wide level. Indeed (and this extends to disciplines other than genomics), any method that uses *a priori* defined regions, or generates a subset of candidate regions prior to inference, is liable to incur a selection bias due to testing hypotheses suggested by the data (14,15). Another option is to exhaustively bin read counts into equally sized tiles across the genome, similar to methods that interrogate other epigenetic marks such as ChIP-Seq and ATAC-Seq (16). However, a bias is incurred when computing the binwise difference of CpG methylation, due to varying numbers and densities of CpG sites within each bin (17).

Ideally, the coordinates of a DMR ought to be called *de novo* from the data at hand, with appropriate FDR controls that are unaffected by pre-screening or other selection biases. Hence the DMR calling process necessitates the application of a heuristic that accounts for both spatial (horizontal) effects, and the actual (vertical) effect of DM. It is for these reasons that, in this study, we conceptualize the CpG site, indexed by a reference genome and represented by sums of methylated and unmethylated reads across both forward and reverse strands, as the fundamental and immutable genomic entity on which DM is evaluated. Subsequently, it follows that a DMR is a *composite* genomic entity that is both bookended by, and summarizes the DM signal across, its constituent CpG sites. We use this principle to guide all simulation and validation methods described henceforth.

The set of available software tools for calling DM is too vast to be described here. Instead, we recommend a number of recent reviews (18–20) of DM calling from WGBS as a good summary of the breadth of available approaches. Two of these (19,20) also perform validation of a selection of tools based on a beta-binomial distribution. Beta-binomial is a popular method for representation and simulation of WGBS data, in that (i) like methylation fraction, the beta component is defined on the [0, 1] interval, (ii) the tendency towards the extremes of this interval can be modelled by the shape parameters α and β, and (iii) the binomial represents discrete methylated and unmethylated read counts. For these reasons, we generate our simulated data under beta-binomial assumptions. However, we do not restrict our suite of methods for benchmarking to those that explicitly assume a beta-binomial distribution of reads, since its compound nature means that *C* and *T* can be represented as separate, marginal negative-binomial or Poisson distributions with different parameters (21,22). Practically, this means that the DM hypothesis can then be represented as an *interaction* effect between a binary *C*/*T* response and the coefficient of interest. It is this observation that motivates our implementation of DMRcate for WGBS data.

In their taxonomy of DM finding methods, both Shafi *et al.* (18) and Huh *et al.* (20) not only explicitly categorize methods by their assumption of beta-binomial data, but also their ability to model WGBS data with covariates. These allow for more complicated study designs and hypotheses, as mentioned previously. In terms of method benchmarking, once we restrict the set of DMR callers to those that both call DMR coordinates *de novo* and incorporate covariates and generalized modelling into their routine, there remain only a small handful.

In eukaryotes, the methylation state of a genomic locus is both cell-type dependent (23) and defined by the genomic sequence context, regulatory and genic features and chromatin state (4,24). However, in each round of replication there is a small degree of maintenance infidelity, leading to a degree of intracellular methylation variability within a succession of CpG sites (25,26), hence it is difficult to define a ‘gold standard’ reference methylome. For the purposes of benchmarking, assuming the methylation fraction of a single locus as ‘fixed’, let alone an entire genome, is a contentious move. Thus, we have taken an empirical approach to characterizing the typical human methylome, based on a large set of consortium-generated data. Though WGBS data simulations are available (21) we decided in favour of implementing our own simulation out of a desire for finer control over specification of α and β. In contrast to other WGBS simulation strategies in the current literature, ours is potentially more realistic in that the parameters are derived from 206 complete human methylomes generated from the BLUEPRINT project (27) as part of the International Human Epigenome Consortium (IHEC). We estimate beta-binomial shape parameters individually for over 26 million CpG sites that have uniquely mapped reference coordinates in GRCh38.p12, creating a library encompassing both population (vertical) and CpG-to-CpG (horizontal) variation of methylation. We then use this library to simulate CpG methylation in order to benchmark both DML and DMR callers. In comparison, Wreczycka *et al.* (19) use fixed values of α and β for their beta-binomially simulated data. Huh *et al.* (20) derive their simulations from biological data but model the proportion of methylated reads from genome-wide methylation fraction means using only a binomial distribution, which does not incorporate the variation between CpGs and across populations as does the beta-binomial. We considered incorporating a local correlation structure into our simulation, as some DMR finders (28,29) explicitly account for this and it has been established to occur in real data (8). However, we decided against this, as for this study we are interested in the effect or coefficient of methylation with respect to a given hypothesis, rather than looking for hidden correlation structures. DMR lengths, however, are informative of correlation and as such we model them based on correlation structure in the BLUEPRINT data.

We also take the opportunity to introduce an expanded version of DMRcate (30), now optimized for DMR calling from both WGBS and Illumina array data and benchmarked against three competing methods (RADmeth (31), dmrseq (29) and DSS (32)). DMRcate’s basic approach for array-based data is that of modelling, via limma (33), logit-transformed methylation fractions and then kernel smoothing the resulting moderated *t*-statistics, with a final step of defining DMRs from an appropriate CpG-level FDR threshold. We find such a strategy is equally applicable, under model specification with an interaction effect, to marginal distributions of log2-transformed methylated and unmethylated WGBS read counts, normalized to total library size (*C* + *T*), and thus we choose this as our favoured implementation.

## MATERIALS AND METHODS

Consistent with our stated concept of DMRs as being an aggregated effect of the differential signal from adjacent CpG sites, we first conducted a benchmarking study of available tools for calling differentially methylated CpGs/loci (DMLs), without reference to their genomic coordinates. We assessed five strategies that meet our stated criteria (see Introduction) to call DMLs under general experimental design (i.e. including a covariate): (i) DSS-general (32), (ii) RADmeth (31), (iii) edgeR (specific to the implementation in Chen *et al.* (2017) (22)), (iv) beta-binomial regression as implemented in the VGAM R package and (v) limma (33) after transformation via *voom* (34).

### A novel application of limma using an interaction effect

The initial step of DMRcate for WGBS is to call DMLs by leveraging various capabilities of the limma package. Our approach closely follows the modelling strategy outlined by Chen *et al.* (22), except that *voom*-fitted log2-transformed counts are used instead of integer counts. In a regular RNA-Seq or microarray experiment there is one measurement for each sample at each genomic location θ. In WGBS data, however, we have two measurements: the read counts *C*_θ_ and *T*_θ_. One option might be to reduce this pair of values to a single value such as $\mathrm{logit}(\frac{C_\theta }{C_\theta + T_\theta }$) so that, again, the data is reduced to a single measurement for each sample at each location. Following the approach of Chen *et al.* (22) however, we analyze the complete per-location data (*C*_θ_, *T*_θ_) as a pair of transformed counts. In this analysis, the library sizes for each sample are used as a GLM offset—that is, a covariate with a fixed (not estimated) coefficient. The intercept term represents overall methylation at the site, and for each covariate, the main effect represents the dependence of the overall methylation level at this location on the covariate, while the interaction between the *C* and *T* counts represents differential methylation. Then empirical Bayes shrinkage can be applied as per usual (35) and per-CpG moderated *t*-statistics and *p*-values are generated.

### Assessing DML detection from WGBS data via simulation

**Figure 1.**
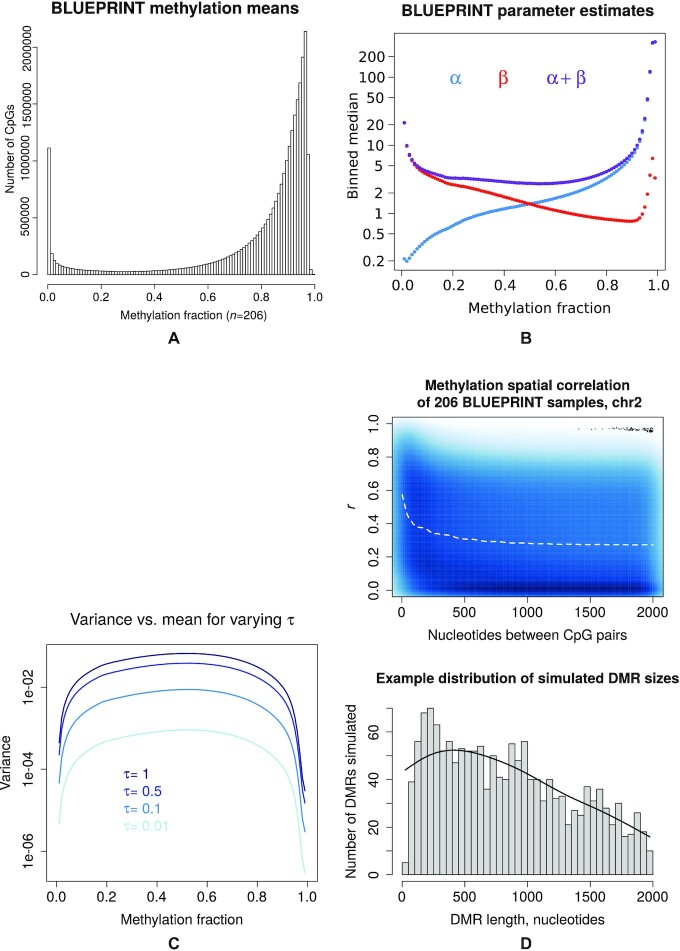
(**A**) Distribution of methylation fraction means of 26 883 210 CpG sites with uniquely mapped reference coordinates over 206 samples from the BLUEPRINT Epigenome Consortium. (**B**) Median estimates of α, β and α + β for 99 non-overlapping bins over the methylation fraction domain for these 206 samples, 0.005 each side of 0.01, 0.02, ..., 0.99. (**C**) Distribution of BLUEPRINT CpG site variances $\frac{\alpha \beta }{(\alpha +\beta )^2(\alpha +\beta +1)}$ when reparameterized by $\tau = \frac{1}{\alpha +\beta }$, for varying values of τ. (**D**) Simulating DMR lengths informed by local correlation of methylation. Above: Density scatterplot of CpG methylation correlation between all methylcytosine pairs within 2kb of each other on chromosome 2, for all 206 BLUEPRINT samples. Methylation fractions are arcsine transformed. Dotted line is a cubic smoothed spline across the domain. Below: Distribution of simulated DMR lengths (in nucleotides) over the same domain.

Benchmarking candidate DML callers requires a data set with a distribution of bisulphite read counts whose parameters are known, which necessitates some degree of simulation. However, we also would ideally like the simulated dataset to closely resemble a set of human methylomes, containing variation appreciably similar to observed data amongst both the DMLs and background CpG sites. Finding a set of parameters that describe the diversity of population distributions of single CpG sites is not trivial. For example, the classic conception of bimodal distribution of methylation fractions across the genome is one of ‘camel humps’, where two peaks tend towards 0 and 1 respectively, but on close inspection of human WGBS data this distribution is asymmetrical, showing a longer, gentler ramp towards the methylated peak than the unmethylated peak (Figure 1 A). Furthermore, this is a global overview of the entire methylome and represents a mixed distribution of multiple methylcytosine loci in each molecule or single cell profile. When this mixture is broken down into single CpG-sites across a population, these peaks are almost always unimodal, tending towards 0 or 1. Rather than arbitrarily selecting parameters to approximate these distributions, we have instead estimated them using public data comprising 206 human samples curated by the BLUEPRINT Epigenome Consortium (27) that have undergone WGBS with a mean coverage between 10x and 100x. These samples comprise 47 different cell types from 5 different tissue sources, both healthy and diseased (Supplementary Table S1). We assumed a beta-binomial distribution of WGBS reads for each individual CpG site, and estimated beta parameters α and β from these 206 samples using the VGAM R package. The beta component of the distribution is described by two shape parameters α and β, with mean $\mu =\frac{\alpha }{\alpha +\beta }$ and variance $V=\frac{\alpha \beta }{(\alpha +\beta )^2(\alpha +\beta +1)}$. Binned distributions of estimates for α and β for given methylation fraction means can be seen in Figure 1 B.

Part of our benchmarking involves testing how the candidate methods respond to different degrees of methylation dispersion. To simplify this concept, we parameterized the beta distribution using μ and $\tau = \frac{1}{\alpha +\beta }$, rather than α and β. For a given methylation fraction μ, $\alpha = \frac{\mu }{\tau }$, $\beta = \frac{1-\mu }{\tau }$ and the variance is $V = \frac{\mu (1-\mu )}{1+\frac{1}{\tau }}$. We can then recover α and β by τ, given μ. The advantage of this reparameterization is that τ now acts as a proxy for variance, and we can then simulate the dispersion of WGBS read counts from a CpG site for multiple values of τ. We can also visualize the overall dispersion trend for the BLUEPRINT Epigenome dataset (Figure 1C), noting that the most variable CpG sites are those with hemimethylated means, and that variance drops away towards the extreme ends of the beta distribution.

To simulate WGBS read counts for CpG sites, we implemented a generative R function (Appendix A) with five variables influencing the resulting dataset. The five variables consisted of (i) *coverage*, Poisson distributed around the mean specified; (ii) absolute *methylation shift* between treatment and control groups in the methylation fraction space; (iii) *sample size* denoting number of treatment/control pairs, as one would for, say, a matched tumour/normal comparison; (iv) *τ* and (v) *covariate size*, implemented as a random patient effect in specified standard deviations from the methylation fraction mean in the logit space. For each instantiation of this function, 100 000 CpG sites were simulated over a paired study design, with *C* and *T* reads generated for both control and treatment arms of each sample. One thousand of these loci (1%) were then earmarked to be differentially methylated. Means for the control group were generated by randomly sampling from the full set of estimated BLUEPRINT means (Figure 1 A). This allowed a heterogeneity of α and β combinations while assessing performance at a given fixed methylation fraction shift, say 0.2. User-specified deviations were applied to all CpGs and groups for the covariate, and then to the flagged DMLs from the treatment group for the methylation shift. The shift was added to the control mean μ if μ ≤ 0.5, and subtracted if μ > 0.5. Values for α and β were generated from the resulting means and given value of τ via lookup from the binned values in Figure 1 B and C. Using these values, the specified coverage count *C* + *T* was randomly split into *C* and *T* reads using the rbetabinom.ab() function from the VGAM R package.

We characterized both first- and second-order effects of these five variables on the performance of the candidate DML detection methods. A range of values was tested for each: coverage ∈ {5×, 10×, 15×, 20×, 30×, 50×, 100×}, methylation shift ∈ {0.01, 0.05, 0.1, 0.2, 0.3, 0.45}, sample size ∈ {3, 5, 10, 15, 20}, τ ∈ {0.01, 0.1, 0.5, 1} and covariate size (in standard deviations) ∈ {0.1, 0.5, 1, 2}. An exhaustive set of combinations was derived from each, resulting in 3360 simulated datasets. Each candidate method was applied to each dataset, hypothesizing a difference in methylation between the matched treatment and control groups, with the pairing covariate included. *P*-values were generated for each of the 100 000 CpG sites, and receiver operating characteristic (ROC) curves were drawn for each simulation. The 1000 DMLs were defined as condition positive, and sensitivity and specificity were defined by the true positive and false positive rates on these. CPU time was also recorded for each candidate method, with all DML calls made on an Intel Xeon E5-2680v3 mapped to a Xenon Radon Duo R1881 cluster node, operating with 24GB RAM.

We also ran an adjunct set of simulations to test the performance of the candidate methods as a function of methylation level itself, based on the above strategy. Fixing coverage at 20x, methylation fraction shift at 0.2, sample size at *n* = 5, τ = 1 and covariate size at 1 s.d., we ran 99 extra simulations of 100 000 CpG sites where the control group methylation fraction mean μ was fixed at 0.01, 0.02, …, 0.99. Like previously, ROCs were drawn for each simulation.

### Assessing DMR detection from WGBS data via simulation

We compared four different self-contained DMR callers with the ability to both call DMRs *de novo* and model generalized experimental hypotheses, as per the criteria outlined in the Introduction: DMRcate (with the aforementioned application of limma) (30), DSS (32), RADmeth (31) and dmrseq (29). Of these, the first three conceptualize DMRs as we outlined earlier: the result of an aggregative process performed *post hoc* to generating per-CpG test statistics. However, dmrseq calls DMRs in a more holistic fashion: calling candidate regions earlier, incorporating correlative information between CpG sites and generating significance values based on comparison to a null via random permutation. The latter two steps are heuristically sensible and so dmrseq’s DMR definition strategy serves as a comparable alternative to our conceptualization.

In order to benchmark tools most adept at DMR discovery and definition, we again aimed to make the simulated dataset as realistic as possible. Four main techniques were adopted in approximating this realism: (i) the set of coordinates that DMRs were called on is precisely the set of GRCh38.p12 reference CpGs; (ii) The DMRs were constructed as sets of successive CpG sites with variable lengths informed by the degree of local methylation correlation within the BLUEPRINT data set; (iii) the non-DMR background CpG sites were taken from a highly homogeneous subset of BLUEPRINT samples and (iv) the constituent CpG sites within a specified DMR were generated by the DML generative function (Appendix A).

All DMR calls were made across a simulated dataset equal in size and CpG coordinate composition to a complete human methylome in order to generate an accurate approximation of real-world hypothesis testing and estimates of CPU resource. DMR coordinates were generated by seeding 3000 start positions at random CpG coordinates in the genome and propagating their length (in successive CpG sites) from that starting point along the forward strand by a gamma distribution with shape=4 and rate=0.2. Propagated loci that overlapped each other, extended past chromosome ends or had a CpG density sparser than 1 per 100 nucleotides were removed prior to benchmarking. While not exact, this resulted in a distribution of DMR lengths resembling the spatial correlation observed over all CpGs sites on chromosome 2 for the full BLUEPRINT WGBS dataset (Figure 1D).

The experimental design for the simulated dataset followed a 5 × 2 structure, where a significant treatment effect was hypothesized across 5 control/treatment pairs of whole methylomes. Constituent CpGs within all DMRs were generated with a methylation shift of 0.2, τ = 1 and a random patient effect of 1 s.d. in logit-space. The remaining non-DMR CpG count data was imported from 10 BLUEPRINT macrophage samples from venous blood (Supplementary Table S2) with a grand mean coverage of 28× across the whole methylome, and these samples were randomized with each data generation. To test the effect of coverage on DMR caller performance, we randomly depleted the read coverage after initial simulation to means of 5×, 10×, 15× and 20×, as well as retaining the original non-depleted 28× coverage simulation.

All four DMR callers were run on each of these five simulations, and ROCs were drawn for each. Germane to our concept of the CpG site as the fundamental unit of differential methylation, and to make the ROCs granular enough to distinguish subtle differences, condition positives were defined as CpGs constitutive of simulated DMR loci and condition negatives as lying outside these loci, to which sensitivity and specificity were defined as the true and false positive rates on these. To make the ROCs as complete as possible, parameters were passed to each DMR caller in order to maximize the methylome range assigned a *p*-value or score. This proved challenging for dmrseq, since minimizing the cutoff parameter—a screening threshold denoting minimum methylation shift for candidate DMRs applied before significance testing takes place—detrimentally influenced both performance and CPU time. Thus, in the interests of fairness we benchmarked dmrseq for multiple values of cutoff. Otherwise, all other default parameters were used for each method, allowing for the paired design specification. The respective tuning parameters used to generate multiple data points on each ROC were the fdr parameter in sequencing.annotate() for DMRcate; the p.threshold parameter in callDMR() for DSS; the -p flag in the dmrs routine for RADmeth and the per-DMR *q*-value for dmrseq. For dmrseq in particular, the complete list of DMRs returned was not enough to draw a complete ROC, so the remainder was imputed linearly to (1, 1). All DMR calls were performed on an Intel Xeon W-2155 Processor with 256GB of RAM.

### Functional enrichment of DMRs

In order to contextualize and validate the biology of DMRs called by the four candidate DMR callers, we again used existing data from the WGBS BLUEPRINT dataset to make comparisons between known cell types. To check whether DMRs were able to characterize B cell biology, we used a subset of three healthy germinal center B cell samples and compared them to three healthy memory B cell samples (Supplementary Table S3). For all four methods, DMRs were thresholded by each routine to produce exactly 2000 DMRs each and were specified to contain a minimum of five CpG sites. Otherwise, default arguments were used. DMRs were then flagged for overlaps with any GeneHancer Double Elite (36) region—a database of known gene regulatory elements with multiple verified sources. The list of corresponding gene names for each overlapping enhancer and/or promoter was then tested for gene set enrichment from the Immunologic ontology from the Molecular Signatures Database (MSigDB) v7.1 (37) using the RITAN Bioconductor package. The background was defined as the complete list of genes with known interactions and promoter regions, and terms with a FDR *q*-value <0.05 were called as significant.

To validate DMRcate DMRs against matched RNA-Seq data, we used a different subset of BLUEPRINT samples, since the B cell subset did not have the full complement of matched transcriptome data. We compared WGBS of five mantle cell lymphoma (MCL) samples to six chronic lymphocytic leukaemia (CLL) samples (Supplementary Table S4). DMRs were called using DMRcate with default parameters and differentially expressed genes (DEGs) were called between these same matched groups of samples using the edgeR glmQLFit() and glmQLFTest() functions (38) with FDR <0.05.

### Implementation of DMRcate for WGBS

The implementation of DMRcate used for this study is version ≥2.0.0, found on Bioconductor release ≥3.10 (https://bioconductor.org/packages/release/bioc/html/DMRcate.html).

## RESULTS

### DML detection

**Figure 2.**
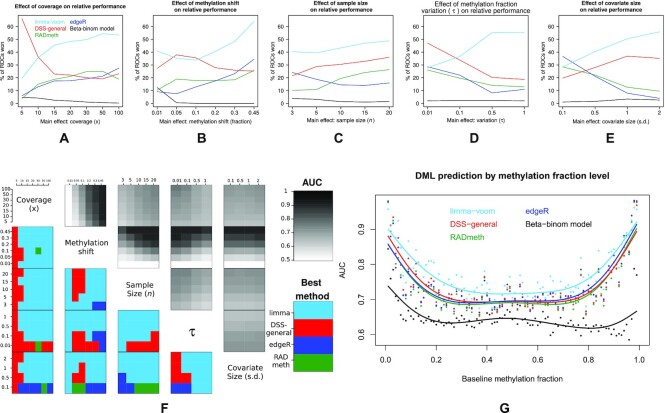
First-order effects of DML benchmarking, measured by percentage of simulations for which a given method incurs the maximum AUC (ties distributed evenly and maximally, hence sums may exceed 100%) for (**A**) coverage, (**B**) absolute methylation shift in the beta space, (**C**) sample size, (**D**) τ and (**E**) covariate size. (**F**) Heatmap of second order joint effects of the variables in (A–E), and the AUC of the winning strategy for those joints. (**G**) Benchmarking performance as a function of mean methylation fraction of control samples. Solid lines are cubic smoothed splines across the domain.

Benchmarking of DML detection methods reveals that relative predictive performance (AUC) is dependent on the nature of the simulated data, as specified by the five variables we modified (see Methods). From the 3360 simulated data sets generated from BLUEPRINT (see Methods), limma performs best on 41% of simulations, DSS-general 27%, RADmeth 15%, edgeR 15% and beta-binomial regression 2% (all rounded to nearest centile) as assessed by area under curve (AUC) from the corresponding ROC (accounting for ties). The best performing strategies as a function of simulated variable value can be viewed in Figure 2A–E. For low coverage (<10×) DMLs, DSS-general is the clear best method, but is overtaken above this value by limma, with edgeR and RADmeth also improving their performance (Figure 2 A). DSS-general is also competitive with limma when the methylation shift is subtle (≤0.1), but limma again becomes dominant when the shift between groups increases (Figure 2 B). This dominance continues across the sample-size domain (Figure 2C), with edgeR showing a relative preference for sample sizes less than 10. Limma also shows a clear superiority as variability (as measured by τ, see Methods) increases, at the expense of all other methods (Figure 2D), and also as the covariate size increases (Figure 2E). Figure 2F shows the second-order joint effects of these five variables, as well as the AUC of the winning strategy. Limma is the clear best performer in the majority of joint cases, with DSS-general superior at the extreme lower end of both coverage and τ, and both edgeR and RADmeth becoming competitive as the covariate size becomes negligible. Unsurprisingly, the degree of methylation shift has the greatest effect on the predictive performance of the best performing strategy, with both coverage and sample size continuing to increase effectiveness at their upper limits (100× and *n* = 20 respectively). Intuitively, the predictive performance increases as τ gets smaller, which implies more neatly separated beta distribution peaks. Surprisingly though, the predictive performance (of limma, at least) shows a subtle increase as the covariate size increases.

When predictive performance is plotted as a function of the base methylation fraction of the control group (Figure 2G), limma again shows superior performance across most of the domain, especially when the methylation fraction is at more intermediate levels. Over the 99 simulations tested, representing base methylation fraction from 0.01, 0.02, …, 0.99, limma incurs the largest AUC in 91 cases, edgeR with 6 and DSS-general with 2.

### FDR control

**Figure 3.**
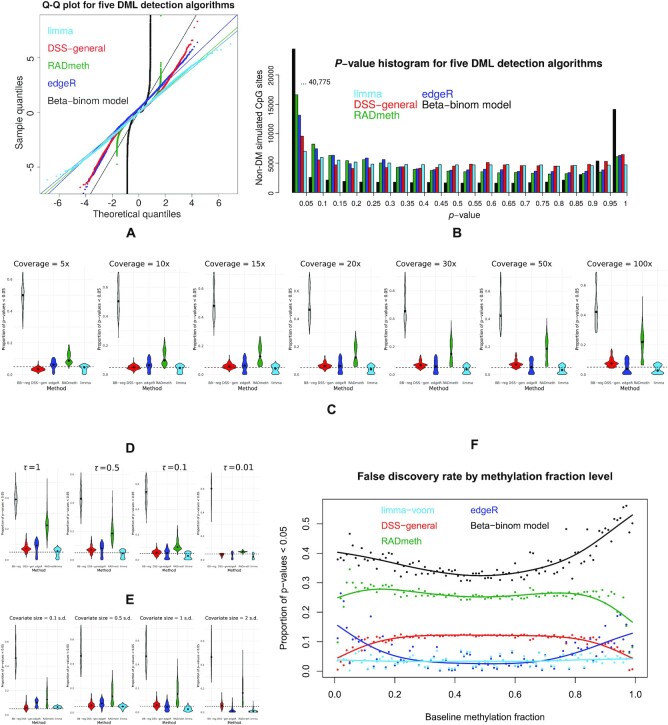
(**A**) *Q*–*Q* plot of *P*-values generated by five DML detection strategies for 99 000 non-DM CpG sites; (**B**) *P*-value histogram from the same set of *P*-values (leftmost black bar truncated). Method-wise proportion of *P*-values <0.05 for non-DM CpGs by (**C**) coverage, (**D**) τ and (**E**) covariate size for the entire set of 3360 simulations. Dashed line at 0.05 represents significance at this level. (**F**) FDR of the five DML detection methods as a function of mean methylation fraction of the control group. Solid lines are cubic smoothed splines across the domain.

The degree to which each DML detection method controls false discovery is seen in Figure 3. We chose a representative simulated dataset (coverage = 20×, methylation shift = 0.2, sample size = 3, τ = 0.5, covariate size = 0.5 s.d.) from our library of simulations derived from BLUEPRINT to show the distribution of *p*-values generated by each DML detection method, for the CpG sites simulated as non-DM (99%). Of all DML detection methods, limma gives the most uniform distribution of *p*-values for non-DM simulated CpG sites. The *Q*–*Q* plot (Figure 3A) clearly shows the distribution of sample quantiles for limma stays very close to the diagonal at the extreme ends, with all other methods straying from the diagonal to varying degrees: edgeR and DSS-general moderately, RADmeth considerably and beta-binomial regression egregiously. This result can also be seen in the corresponding *p*-value histogram (Figure 3B), with limma showing the most uniformity towards both 0 and 1. This uniformity is consistent regardless of coverage (Supplementary Figure 1A). Investigating further, we find that beta-binomial regression is highly oversensitive under all conditions, and that RADmeth is also susceptible to increasing proportions of false positives as the coverage increases (Figure 3C). A similar scenario appears when τ is increased (Figure 3D). Increasing the covariate size results in quite different responses for each method. Limma and edgeR become more conservative in their FDR estimation, tending towards false negatives, whereas RADmeth increases its false positive rate, and DSS-general is highly consistent across the domain (Figure 3E). Both methylation shift and sample size have very little effect on FDR control patterns (Supplementary Figure S1B and C). The adjunct simulation assessing performance as a function of methylation fraction shows also shows limma maintaining a low and consistent FDR across the entire domain (Figure 3F), in contrast to the other methods whose FDR is influenced by the extremities of the domain to a far greater degree.

### Computational time: DML calling

**Figure 4.**
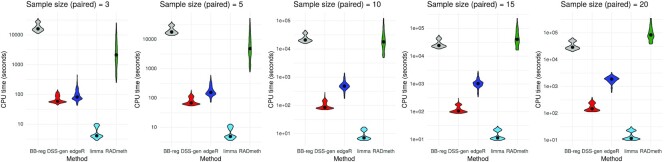
Serial CPU time taken by the five candidate DML callers for all 3360 simulations as a function of sample size.

It is clear that limma outperforms other DML callers both in terms of predictive performance and computational time. Calculating *P*-values for 100 000 CpG sites, limma was fastest for every single simulation, taking 10.39 s on average (Figure 4) in serial time. The next fastest was DSS-general with 1 min 50 s, then edgeR with 12 min 28 s, beta-binomial regression ≈7 h and RADmeth ≈11 h. The only simulated variable with an appreciable effect on the CPU time needed was sample size, whose increase penalized the candidate methods at markedly different rates. CPU time as a function of the other four variables can be viewed in Supplementary Figure S2.

### DMR detection

**Figure 5.**
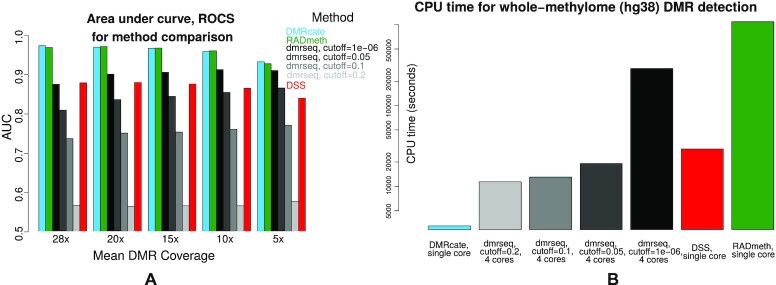
(**A**) Predictive performance of various DMR callers on simulated WGBS data for various coverage values. (**B**) Serial CPU time (unless otherwise specified) required by each caller for the non-depleted (28×) dataset.

DMR caller benchmarking was performed on four strategies described in the Methods section. For all coverage values tested, DMRcate and RADmeth are the two best predictive strategies, with <0.01 AUC difference between them in each case (Figure 5 A). DSS and dmrseq fare less well, with the screening threshold value cutoff having a marked effect on dmrseq’s DMR detection ability. Despite a simulated methylation shift of 0.2 for all DMRs, a progressive decrease of cutoff below this value seems to allow more DMRs to be called as true positive by dmrseq, mitigating the need for large sections of the ROC to be imputed (Supplementary Figure S3A). A recapitulation of the data in Figure 5A, grouped by method, can be viewed in Supplementary Figure S3B.

As extra confirmation for the default settings of DMRcate, we also benchmarked various kernel sizes (controlled by the value of parameter *C* in the call to dmrcate()) to determine whether the optimal size for WGBS data differs to that of Illumina arrays (30) on the same simulations. Encouragingly, and perhaps surprisingly, we found that the default kernel size (500 bp = 1 s.d. of kernel support, i.e. *C* = 2 and λ = 1000) is optimal (Supplementary Figure S3C and S3D), suggesting that this width reflects a spatial correlation of DNA methylation consistent with underlying biology, rather than it necessarily being an artefact of the measuring platform.

### Computational time: DMR calling

We observed large differences in computational time between DMR callers from these simulations. The CPU time for the non-depleted simulation is shown in Figure 5B. CPU times are reported as the entire time taken for the set of routines needed to call DMRs from the CpG-wise input of *C* and *T* counts; in other words the DML calling routine (if applicable) is included in the total time. Serial time is reported except for dmrseq, where the recommended use of four CPU cores was specified. DMRcate is clearly the fastest DMR caller, with DMRs able to be called from a set of complete human methylomes in serial under one hour. dmrseq’s performance improves as cutoff increases, but as described earlier this comes at the expense of predictive power. Despite RADmeth’s excellent predictive capability at calling DMRs, they are called very slowly; this time is almost entirely taken up by the DML calling step. This step can be easily parallelized, but the user would then need over 300 cores to match DMRcate’s serial CPU time.

### Minimum required coverage

We observe a non-linear relationship between WGBS coverage and predictive performance for both DMLs and DMRs (Figures 2F, 5A, Supplementary Figure S3B). Intuitively, the minimum coverage needed to detect DM depends on the size of the methylation shift, but for a subtle shift such as 0.2, no plateau is observed. Gains are certainly made increasing the coverage from the lower end of the domain, but the relative increase in terms of DMR detection begins to flatten above 15×. Counter-intuitively, the predictive performance of dmrseq worsens as the coverage is increased, which is likely the result of decreased specificity.

### Functional enrichment of DMRs

**Figure 6.**
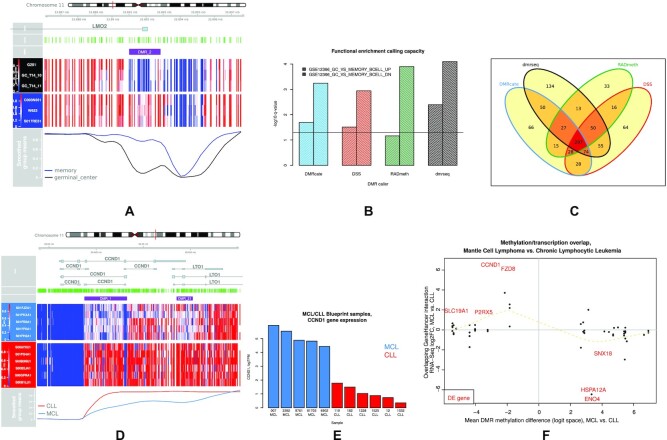
(**A**) A DMR (generated by the DMR.plot() function in DMRcate) hypomethylated in germinal center B cells compared to normal memory B cells over the LMO2 promoter region. (**B**) Significance from functional enrichment tests from the four candidate DMR callers of target MSigDB immunologic terms GC_VS_MEMORY_BCELL_UP and GC_VS_MEMORY_BCELL_DN when comparing germinal center B cells to normal memory B cells. (**C**) Venn diagram describing overlap of significant (FDR *q*-value < 0.05) immunologic MSigDB immunologic terms. (**D**) A DMR hypomethylated in MCL tumour cells compared to CLL tumour cells over the CCND1 locus. (**E**) Normalized gene expression values for CCND1 in these same samples. (**F**) Integration of DMRs and gene expression values via overlapping GeneHancer Double Elite regulatory regions. DEGs are shown in red and in lieu of dots. Dotted line is a cubic smoothed spline across the domain.

The biological relevance of DMRs called by the four candidate DMR callers was validated by comparing germinal center B cells to normal memory B cells using correspondent BLUEPRINT samples (Supplementary Tables S3 and S5). Genes activated by known regulatory regions overlapping these DMRs are enriched for terms consonant with the underlying biology. For example, the 2nd most significant genome-wide DMR called by DMRcate is positioned directly over the LMO2 promoter (Figure 6A), which is a known germinal center marker (39). The target MSigDB immunologic ontology terms GC_VS_MEMORY_BCELL_DN and GC_VS_MEMORY_BCELL_UP were both called as significant (FDR *q*-value <0.05) by all four candidate methods, except GC_VS_MEMORY_BCELL_UP by RADmeth which only marginally fell below significance. Dmrseq called both these terms with the most significant *q*-value (Figure 6B). However, dmrseq is also the outlier when the total number of terms called is taken into account (Figure 6C), uniquely calling 134 off-target terms as significantly enriched. This is likely because dmrseq’s default settings generally call longer DMRs, but more broadly this indicates a tradeoff between sensitivity and specificity inherent in functional enrichment tests.

Validation of DMRcate DMRs via matched RNA-Seq samples was achieved by comparing mantle cell lymphoma (MCL) to chronic lymphocytic leukaemia (CLL) using correspondent BLUEPRINT samples (Supplementary Table S4). A hallmark feature and driver mutation in MCL tumour cells is a translocation event resulting in the overexpression of CCND1 (40). DMRcate identified the top DMR between MCL and CLL as the hypomethylation of the CCND1 locus in MCL samples (Figure 6D, Supplementary Table S6). Concurrently, CCND1 was confirmed to be the most significantly upregulated gene for the same comparison for matched RNA-Seq data (Figure 6E, Supplementary Table S7). A possible explanation for this upregulation is that the translocation interferes with regular epigenetic silencing of CCND1. We extended this hypothesis to the full set of DMRs called, integrating the methylation data with the gene expression data by plotting the corresponding gene expression fold changes (connected via DMR overlap with GeneHancer Double Elite regulatory regions) against the methylation shift (Figure 6F). All differentially expressed genes (FDR < 0.05) appear in the second and fourth quadrants of this plot, indicating that DNA methylation may play a pivotal role as a mediator of distinguishing transcriptional profiles for these two tumour types via silencing of promoter and enhancer regions.

## DISCUSSION

The superior predictive performance of DMR callers DMRcate and RADmeth suggest that DMR detection is best served by a two-step heuristic favoured by both methods: first generate test statistics for individual CpG site data over the entire corpus of measured CpGs, and then aggregate these results to form DMRs with appropriate FDR controls as a final step. This contrasts with methods such as dmrseq and bumphunter (41) where the aggregation step is performed further upstream, followed by significance testing using permutation. However, the risk with upstream aggregation and candidate locus definition is that it may act as a functional localizer, thus incurring a selection bias or ‘double dipping’, since thresholding is applied to the candidate regions twice. This issue has been discussed at length with regards to the detection of localized hotspots from functional MRI studies (14,42). In the context of DM, the CpG site is equivalent to a voxel and the DMR equivalent to a cluster of voxels. The suggested remedy is, if possible, to perform exhaustive inference over the complete domain before defining regions of interest (ROI), which is the strategy implemented by DMRcate, RADmeth and DSS.

It follows that the performance of the DMR caller hinges substantially on the predictive performance of the initial inference, which has been demonstrated by our benchmarking of DML callers. The superior performance of our novel application of limma is likely down to three characteristics: (i) the explicit normalization of WGBS counts to library size, (ii) the lowess mean-variance fit via *voom* and (iii) empirical Bayes variance shrinkage. By comparison, the edgeR implementation we have tested does not possess characteristic (ii), although it is possible within alternative edgeR workflows to estimate a non-parametric mean-variance trend analogous to limma. RADmeth has no normalization step, and only a common dispersion parameter coded into its beta-binomial regression rather than a trended dispersion like in edgeR and limma, both of which may explain its tendency to give false positives under higher coverage scenarios. DSS-general takes a different approach altogether, using an arcsine transformation of methylation fractions and estimation of the dispersion parameter using Pearson’s χ^2^ under beta-binomial assumptions. This works well with respect to FDR control and is superior to limma in the case of low (<10×) coverage scenarios. It is for this reason that we allow the user the option of using DSS-general as an alternative to limma to generate per-CpG test statistics for DMRcate. VGAM’s beta-binomial regression applies none of the aforementioned strategies and likely suffers as a result.

The decreasing variance *V* of the beta-binomial the distribution as the mean μ tends towards the methylation fraction extremes of 0 and 1 (Figure 1C) influences the performance of each DML caller. All methods improve their performance the closer μ gets to 0 or 1 (Figure 2G), which is unsurprising given the decrease in dispersion. Less expected is their handing of the FDR. False discoveries from edgeR and beta-binomial regression are increased towards these extremes, while they are decreased under RADmeth and DSS-general. Only our novel application of limma appears to be invariant to μ. The ramification of this is that DMRcate, with this application of limma calling DMLs as a primary step, is able to standardize the FDR across the methylation domain without preferentially calling DMRs towards or away from its extremes.

The aggregation strategies and FDR control are quite different for each DMR caller. DMRcate employs dynamic thresholding where the total number of constituent CpGs for all DMRs is indexed by the number of significant individual CpG sites at the specified FDR. This approach is inherently conservative and prioritizes the minimization of Type I errors but can be easily adjusted by relaxing the initial FDR at which significantly DM CpGs are called. DSS’ approach is relatively simple, merging proximal DMLs and defining DMRs by providing a lower bound on the percentage of CpG sites that are DMLs. RADmeth’s aggregation is more sophisticated in that it adjusts the per-CpG *P*-values based on how they correlate with neighbours using a Stouffer–Liptak test, which is the approach of comb-p (43). The result in this study squares with our previous benchmarking of comb-p against DMRcate (30) where we found both methods had comparable predictive performance. However, we still recommend using DMRcate over RADmeth for a number of reasons. Firstly, the CPU resource required for RADmeth is over two orders of magnitude greater than DMRcate (Figure 5B). Secondly, RADmeth’s DMRs may be more permissively defined due to the *p*-value inflation of the DML caller (Figure 3A, B and F). Lastly, DMRcate is implemented in R and maintained on Bioconductor, which allows seamless integration with other genomic workflows, and contains additional functionality such as visualization (Figure 6A and D).

In addition, DMRcate can model any factorial or non-factorial design able to be parsed by limma. This gives it an advantage over other DMR callers in that it can test more complex experimental designs, such as those with post-hoc contrasts and/or interaction effects. Table 1 describes the ability of the DMR callers we have tested to perform inference over various experimental setups. DMRcate, along with DSS, is the most versatile for model specification. This, combined with its superior predictive performance to DSS and dmrseq, aforementioned advantages over RADmeth while matching its predictive performance, and other aspects such as accessibility and DMR visualization, represents a major improvement on existing methodology.

**Table 1.** Factorial design capabilities of the four DMR callers tested

DMR caller experimental design capabilities				
	DMRcate	DSS	RADmeth	dmrseq
Paired design	✓	✓	✓	✓
Covariates	✓	✓	✓	✓
Continuous response	✓	✓	✓	✓
Post-hoc contrasts	✓	✓	✗	✗
Interaction effects	✓	✓	✓	✗

In terms of answering the practical question of the minimum amount of coverage needed to call DMRs, we do not see an obvious plateau when absolute methylation shifts are subtle (0.2). This is in line with previous work validating the reproducibility of WGBS measurements as a function of coverage (44). For detection of absolute differences >0.3, a mean whole genome coverage of 15× is likely sufficient, and above this depth detection gains tend to gradually diminish.

Our simulated dataset is unique in that it is begotten from a systematic catalogue of sampled DNA methylation variation amongst human cells, with identical scale to the human genome and high granularity at CpG-level resolution. This high level of evocation allows for a realistic appraisal of tools for detecting DNA methylation. One limitation of our approach is that the 206 BLUEPRINT samples used are highly enriched for haematopoietic lineages, and so the results herein may not be reproducible on tissues that differ substantially from blood in their methylation profile. However, our suite of simulations is diversified as a result of varying τ from our parameterization of the beta distribution, whose extensions may bear similarities to other tissues.

## CONCLUSION

The benchmarking and comparisons contained herein represent a desire to motivate discussion about how we define genomic phenomena. We have demonstrated that the preferable strategy for defining DMRs is to construct them by aggregating the differential signal from individual CpG sites, leading to a conception of DMRs as a composite genomic entity rather than one that is self-contained and immutable. It is with this in mind that we present DMRcate as a flexible, accurate and accessible DMR caller, and our benchmarking finds it at or exceeding competing best practice.

## DATA AVAILABILITY

The datasets analysed in this study are available in the BLUEPRINT repository, http://ftp.ebi.ac.uk/pub/databases/blueprint/data/homo_sapiens/GRCh38/. DMRcate is maintained at https://github.com/timpeters82/DMRcate-devel.

## Supplementary Material

gkab637_Supplemental_Files

## References

[1] Bird A.P. CpG-rich islands and the function of DNA methylation. Nature. 1986; 321:209–213. 2423876 10.1038/321209a0

[2] Suzuki M.M. , Bird A. DNA methylation landscapes: provocative insights from epigenomics. Nat. Rev. Genet. 2008; 9:465–476. 18463664 10.1038/nrg2341

[3] Greenberg M.V. , Bourc’his D. The diverse roles of DNA methylation in mammalian development and disease. Nat. Rev. Mol. Cell Biol. 2019; 20:590–607. 31399642 10.1038/s41580-019-0159-6

[4] Jones P.A. Functions of DNA methylation: islands, start sites, gene bodies and beyond. Nat. Rev. Genet. 2012; 13:484–492. 22641018 10.1038/nrg3230

[5] Baylin S.B. , Jones P.A. A decade of exploring the cancer epigenome-biological and translational implications. Nat. Rev. Cancer. 2011; 11:726–734. 21941284 10.1038/nrc3130 PMC3307543

[6] Clark S.J. , Harrison J., Paul C.L., Frommer M. High sensitivity mapping of methylated cytosines. Nucleic Acids Res. 1994; 22:2990–2997. 8065911 10.1093/nar/22.15.2990 PMC310266

[7] Clark S. , Statham A., Stirzaker C., Molloy P., Frommer M. DNA methylation: bisulphite modification and analysis. Nat. Protoc. 2006; 1:2353–2364. 17406479 10.1038/nprot.2006.324

[8] Eckhardt F. , Lewin J., Cortese R., Rakyan V.K., Attwood J., Burger M., Burton J., Cox T.V., Davies R., Down T.A. et al. DNA methylation profiling of human chromosomes 6, 20 and 22. Nat. Genet. 2006; 38:1378–1385. 17072317 10.1038/ng1909 PMC3082778

[9] Lister R. , Pelizzola M., Dowen R.H., Hawkins R.D., Hon G., Tonti-Filippini J., Nery J.R., Lee L., Ye Z., Ngo Q.-M. et al. Human DNA methylomes at base resolution show widespread epigenomic differences. Nature. 2009; 462:315–322. 19829295 10.1038/nature08514 PMC2857523

[10] Tukey J.W. Comparing individual means in the analysis of variance. Biometrics. 1949; 5:99. 18151955

[11] Feil R. , Handel M.A., Allen N.D., Reik W. Chromatin structure and imprinting: developmental control of DNase-I sensitivity in the mouse insulin-like growth factor 2 gene. Dev. Genet. 1995; 17:240–252. 8565330 10.1002/dvg.1020170309

[12] Li W. , Bernaola-Galván P., Haghighi F., Grosse I. Applications of recursive segmentation to the analysis of DNA sequences. Comput. Chem. 2002; 26:491–510. 12144178 10.1016/s0097-8485(02)00010-4

[13] Klein H.-U. , Hebestreit K. An evaluation of methods to test predefined genomic regions for differential methylation in bisulfite sequencing data. Brief. Bioinform. 2016; 17:796–807. 26515532 10.1093/bib/bbv095

[14] Friston K. , Rotshtein P., Geng J., Sterzer P., Henson R. A critique of functional localisers. NeuroImage. 2006; 30:1077–1087. 16635579 10.1016/j.neuroimage.2005.08.012

[15] Benjamini Y. Simultaneous and selective inference: current successes and future challenges. Biometrical J. 2010; 52:708–721. 10.1002/bimj.200900299 21154895

[16] Lun A.T. , Smyth G.K. csaw: a Bioconductor package for differential binding analysis of ChIP-seq data using sliding windows. Nucleic Acids Res. 2016; 44:e45. 26578583 10.1093/nar/gkv1191 PMC4797262

[17] Singer M. , Pachter L. Controlling for conservation in genome-wide DNA methylation studies. BMC Genomics. 2015; 16:420. 26024968 10.1186/s12864-015-1604-3 PMC4448855

[18] Shafi A. , Mitrea C., Nguyen T., Draghici S. A survey of the approaches for identifying differential methylation using bisulfite sequencing data. Brief. Bioinform. 2018; 19:737–753. 28334228 10.1093/bib/bbx013 PMC6171488

[19] Wreczycka K. , Gosdschan A., Yusuf D., Grüning B., Assenov Y., Akalin A. Strategies for analyzing bisulfite sequencing data. J. Biotechnol. 2017; 261:105–115. 28822795 10.1016/j.jbiotec.2017.08.007

[20] Huh I. , Wu X., Park T., Yi S.V. Detecting differential DNA methylation from sequencing of bisulfite converted DNA of diverse species. Brief. Bioinform. 2019; 20:33–46. 28981571 10.1093/bib/bbx077 PMC6357555

[21] Rackham O. J.L. , Dellaportas P., Petretto E., Bottolo L. WGBSSuite: simulating whole genome bisulphite sequencing data and benchmarking differential DNA methylation analysis tools. Bioinformatics. 2015; 31:2371–2373. 25777524 10.1093/bioinformatics/btv114 PMC4495289

[22] Chen Y. , Pal B., Visvader J.E., Smyth G.K. Differential methylation analysis of reduced representation bisulfite sequencing experiments using edgeR. F1000Research. 2017; 6:2055. 29333247 10.12688/f1000research.13196.1 PMC5747346

[23] Reik W. , Dean W., Walter J. Epigenetic reprogramming in mammalian development. Science. 2001; 293:1089–1093. 11498579 10.1126/science.1063443

[24] Onuchic V. , Lurie E., Carrero I., Pawliczek P., Patel R.Y., Rozowsky J., Galeev T., Huang Z., Altshuler R.C., Zhang Z. et al. Allele-specific epigenome maps reveal sequence-dependent stochastic switching at regulatory loci. Science. 2018; 361:eaar3146. 30139913 10.1126/science.aar3146 PMC6198826

[25] Du Q. , Bert S.A., Armstrong N.J., Caldon C.E., Song J.Z., Nair S.S., Gould C.M., Luu P.-L., Peters T., Khoury A. et al. Replication timing and epigenome remodelling are associated with the nature of chromosomal rearrangements in cancer. Nat. Commun. 2019; 10:416. 30679435 10.1038/s41467-019-08302-1 PMC6345877

[26] Riggs A.D. DNA methylation and late replication probably aid cell memory, and type I DNA reeling could aid chromosome folding and enhancer function. Philos. T. Roy. Soc. B. 1990; 326:10.1098/rstb.1990.0012. 1968665

[27] Stunnenberg H.G. , International Human Epigenome Consortium S., Hirst M., de Almeida M., Altucci L., Amin V., Amit I., Antonarakis S.E., Aparicio S., Arima T. et al. The International Human Epigenome Consortium: a blueprint for scientific collaboration and discovery. Cell. 2016; 167:1145–1149. 27863232 10.1016/j.cell.2016.11.007

[28] Sofer T. , Schifano E.D., Hoppin J.A., Hou L., Baccarelli A.A. A-clustering: a novel method for the detection of co-regulated methylation regions, and regions associated with exposure. Bioinformatics. 2013; 29:2884–2891. 23990415 10.1093/bioinformatics/btt498 PMC3810849

[29] Korthauer K. , Chakraborty S., Benjamini Y., Irizarry R.A. Detection and accurate false discovery rate control of differentially methylated regions from whole genome bisulfite sequencing. Biostatistics. 2018; 20:367–383. 10.1093/biostatistics/kxy007 PMC6587918 29481604

[30] Peters T. , Buckley M., Statham A., Pidsley R., Samaras K., Lord R., Clark S., Molloy P. De novo identification of differentially methylated regions in the human genome. Epigenet. Chromatin. 2015; 8:6. 10.1186/1756-8935-8-6 PMC4429355 25972926

[31] Dolzhenko E. , Smith A.D. Using beta-binomial regression for high-precision differential methylation analysis in multifactor whole-genome bisulfite sequencing experiments. BMC Bioinformatics. 2014; 15:215. 24962134 10.1186/1471-2105-15-215 PMC4230021

[32] Park Y. , Wu H. Differential methylation analysis for BS-seq data under general experimental design. Bioinformatics. 2016; 32:1446–1453. 26819470 10.1093/bioinformatics/btw026 PMC12157722

[33] Ritchie M.E. , Phipson B., Wu D., Hu Y., Law C.W., Shi W., Smyth G.K. limma powers differential expression analyses for RNA-sequencing and microarray studies. Nucleic Acids Res. 2015; 43:e47. 25605792 10.1093/nar/gkv007 PMC4402510

[34] Law C.W. , Chen Y., Shi W., Smyth G.K. voom: precision weights unlock linear model analysis tools for RNA-seq read counts. Genome Biol. 2014; 15:R29. 24485249 10.1186/gb-2014-15-2-r29 PMC4053721

[35] Smyth G.K. Linear models and empirical Bayes methods for assessing differential expression in microarray experiments. Stat. Appl. Genet. Mo. B. 2004; 3:Article3. 10.2202/1544-6115.1027 16646809

[36] Fishilevich S. , Nudel R., Rappaport N., Hadar R., Plaschkes I., Iny Stein T., Rosen N., Kohn A., Twik M., Safran M. et al. GeneHancer: genome-wide integration of enhancers and target genes in GeneCards. Database. 2017; 2017:bax028. 10.1093/database/bax028 PMC5467550 28605766

[37] Subramanian A. , Tamayo P., Mootha V.K., Mukherjee S., Ebert B.L., Gillette M.A., Paulovich A., Pomeroy S.L., Golub T.R., Lander E.S. et al. Gene set enrichment analysis: A knowledge-based approach for interpreting genome-wide expression profiles. Proc. Natl. Acad. Sci. U.S.A. 2005; 102:15545–15550. 16199517 10.1073/pnas.0506580102 PMC1239896

[38] Lun A.T. , Chen Y., Smyth G.K. It’s DE-licious: a recipe for differential expression analyses of RNA-seq experiments using quasi-likelihood methods in edgeR. Methods in Molecular Biology. 2016; 1418:10.1007/978-1-4939-3578-9_19 27008025

[39] Natkunam Y. , Zhao S., Mason D.Y., Chen J., Taidi B., Jones M., Hammer A.S., Dutoit S.H., Lossos I.S., Levy R. The oncoprotein LMO2 is expressed in normal germinal-center B cells and in human B-cell lymphomas. Blood. 2007; 109:1636–1642. 17038524 10.1182/blood-2006-08-039024 PMC1794056

[40] Jares P. , Colomer D., Campo E. Genetic and molecular pathogenesis of mantle cell lymphoma: perspectives for new targeted therapeutics. Nat. Rev. Cancer. 2007; 7:750–762. 17891190 10.1038/nrc2230

[41] Jaffe A.E. , Murakami P., Lee H., Leek J.T., Fallin M.D., Feinberg A.P., Irizarry R.A. Bump hunting to identify differentially methylated regions in epigenetic epidemiology studies. Int. J. Epidemiol. 2012; 41:200–209. 22422453 10.1093/ije/dyr238 PMC3304533

[42] Kriegeskorte N. , Simmons W.K., Bellgowan P.S., Baker C.I. Circular analysis in systems neuroscience: The dangers of double dipping. Nat. Neurosci. 2009; 12:535–540. 19396166 10.1038/nn.2303 PMC2841687

[43] Pedersen B.S. , Schwartz D.A., Yang I.V., Kechris K.J. Comb-p: software for combining, analyzing, grouping and correcting spatially correlated P-values. Bioinformatics. 2012; 28:2986–2988. 22954632 10.1093/bioinformatics/bts545 PMC3496335

[44] Peters T.J. , French H.J., Bradford S.T., Pidsley R., Stirzaker C., Varinli H., Nair S., Qu W., Song J., Giles K.A. et al. Evaluation of cross-platform and interlaboratory concordance via consensus modelling of genomic measurements. Bioinformatics. 2019; 35:560–570. 30084929 10.1093/bioinformatics/bty675 PMC6378945

