# Supplementary material for: Calling differentially methylated regions from whole genome bisulphite sequencing with DMRcate
Source: Nucleic Acids Res. 2021 Jul 28;49(19):e109. doi: 10.1093/nar/gkab637 (PMC8565305; doi:10.1093/nar/gkab637)
Supplement: gkab637_Supplemental_Files [file gkab637_supplemental_files.zip › Appendix_A.pdf]

Appendix A: Calling differentially methylated regions from whole genome bisulphite sequencing with DMRcate

Timothy J. Peters, Michael J. Buckley, Yunshun Chen, Gordon K. Smyth, Christopher C. Goodnow and Susan J. Clark

May 26, 2021

The R code below was used to generate simulated WGBS data for benchmarking of both DML and DMR callers. The `blueprint_means` and `estimates` objects are available on request.

```
require(VGAM)

generate_count_matrix <- function(blueprint_means,
                                  estimates,
                                  coverage_mean,
                                  shift,
                                  samples,
                                  bioreps,
                                  vif,
                                  latent_sd,
                                  numloci=100000){

  stopifnot(coverage_mean > 0)
  stopifnot(shift > 0 & shift < 0.5)
  stopifnot(samples > 1)
  stopifnot(length(shift) == bioreps-1)

  #Sample real methylation means from the logit-transformed blueprint dataset
  controlmean <- sample(blueprint_means, numloci, replace=F)
  hyper <- controlmean > 0

  # Randomly select 1% of loci to be differentially methylated (DM)
  DM_index <- sample(1:numloci, round(numloci/100, 0), replace = F)
  hyper_treat <- hyper[DM_index]

  # Generate random effect for patient/sample
  deviations <- rnorm(samples, 0, latent_sd)
  ctrl_effect <- t(sapply(controlmean, function (x) x + deviations))

  #Replicate and backtransform
  controlmeans <- matrix(ctrl_effect, nrow=nrow(ctrl_effect),
                         ncol=samples*bioreps)
  fin_means <- VGAM::logit(controlmeans, inverse = T)

  #Generate treatment effect
  # Each group of bioreps (i.e. phenotype) has a shift FROM the control
  for(i in 1:(bioreps-1)){
    fin_means[DM_index,][hyper_treat,(samples*i + 1):((i+1)*samples)] <-
      fin_means[DM_index,][hyper_treat,(samples*i + 1):((i+1)*samples)] - shift[i]
```

```
    fin_means[DM_index,][!hyper_treat,(samples*i + 1):((i+1)*samples)] <-
      fin_means[DM_index,][!hyper_treat,(samples*i + 1):((i+1)*samples)] + shift[i]
  }

  #Adjust for extreme values
  fin_means[fin_means < 0.01] <- 0.01
  fin_means[fin_means > 0.99] <- 0.99

  #Round both to nearest centile
  fin_means <- round(100*fin_means, 0)/100

  #Then grab estimates of alpha and beta from our binned data in estimates

  alphas <- fin_means/(vif*estimates$tau[match(fin_means, estimates$mean)])
  betas <- (1-fin_means)/(vif*estimates$tau[match(fin_means, estimates$mean)])

  #Generate counts for all columns

  coverage <- rpois(numloci*ncol(alphas), coverage_mean)
  C_counts <- VGAM::rbetabinom.ab(numloci*ncol(alphas), size = coverage,
                                  shape1 = alphas, shape2 = betas)
  C_counts <- matrix(C_counts, nrow=numloci, ncol=ncol(alphas))
  T_counts <- coverage - C_counts

  countmatrix <- eval(parse(text=paste0("cbind(", paste(sapply(1:ncol(C_counts),
                function (x) gsub("idx", x, "C_counts[,idx], T_counts[,idx]")),
                                  collapse=', '), ")")))

  colnames(countmatrix) <- paste(rep(c(paste0("Ctrl_", 1:samples),
                                       paste(rep(paste0("Treat", 1:(bioreps-1)),
                                       each=samples), 1:samples, sep="_")), each=2),
                                       c("C", "T"), sep=".")
  rownames(countmatrix) <- paste("NULL", 1:nrow(countmatrix), sep="_")
  rownames(countmatrix)[DM_index] <- paste("DM", 1:length(DM_index), sep="_")
  countmatrix
}
```
