# Supplementary material for: Calling differentially methylated regions from whole genome bisulphite sequencing with DMRcate
Source: Nucleic Acids Res. 2021 Jul 28;49(19):e109. doi: 10.1093/nar/gkab637 (PMC8565305; doi:10.1093/nar/gkab637)
Supplement: gkab637_Supplemental_Files [file gkab637_supplemental_files.zip › Supplementary.pdf]

Supplementary Material: Calling differentially methylated regions from whole genome bisulphite sequencing with DMRcate

Timothy J. Peters, Michael J. Buckley, Yunshun Chen, Gordon K. Smyth, Christopher C. Goodnow and Susan J. Clark

Supplementary Tables

Supplementary Tables 1-7 can be found in `Supplementary_Table_[1-7].xlsx`.

- Supplementary Table 1: Metadata for the complete set of 206 BLUEPRINT WGBS samples used for estimating beta-binomial parameters for simulation.
- Supplementary Table 2: Metadata for 10 BLUEPRINT WGBS samples from macrophages used as whole genome background for the simulated datasets used to benchmark DMR callers.
- Supplementary Table 3: Metadata for 6 BLUEPRINT WGBS samples used for gene ontology enrichment of candidate DMR callers.
- Supplementary Table 4: Metadata for 11 BLUEPRINT samples used for integration analysis of differential methylation and differential expression between mantle cell lymphoma (MCL) and chronic lymphocytic leukaemia (CLL).
- Supplementary Table 5: DMRs called by all four candidate DMR callers for germinal center vs. memory B cell samples in Supplementary Table 3.
- Supplementary Table 6: DMRs called by DMRcate for MCL vs. CLL samples in Supplementary Table 4.
- Supplementary Table 7: edgeR `topTags()` output for RNA-Seq differential expression hypothesised between MCL and CLL samples in Supplementary Table 4.

Supplementary Figures

Supplementary Figure 1: **(a)** *P*-value histogram of limma results for non-DM CpGs as WGBS coverage is simulated at various levels. All parameters are identical to those set for Figure 3b. Method-wise proportion of *p*-values < 0.05 for non-DM CpGs by **(b)** methylation shift and, **(c)** sample size size for the entire set of 3,360 simulations.

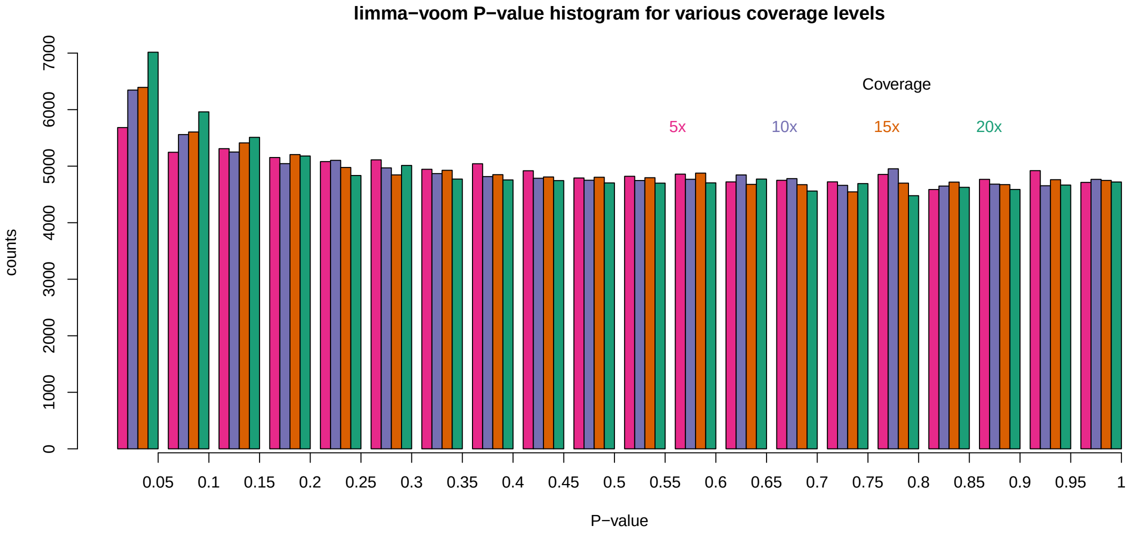

(a)

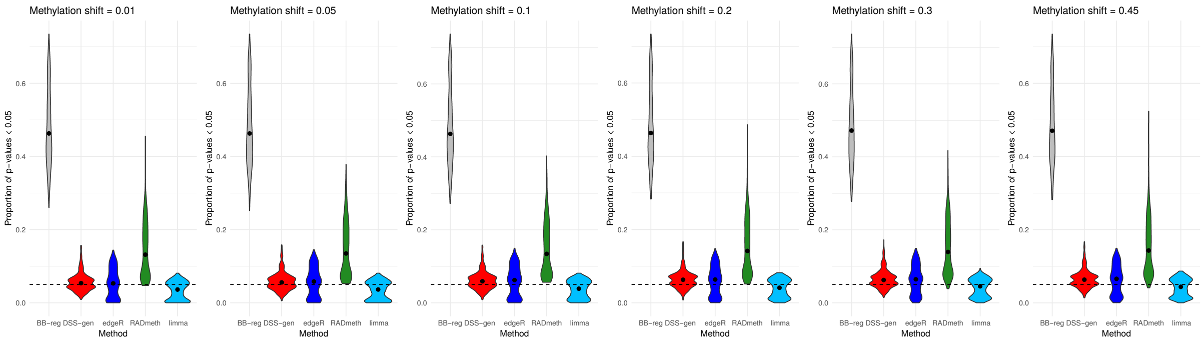

(b)

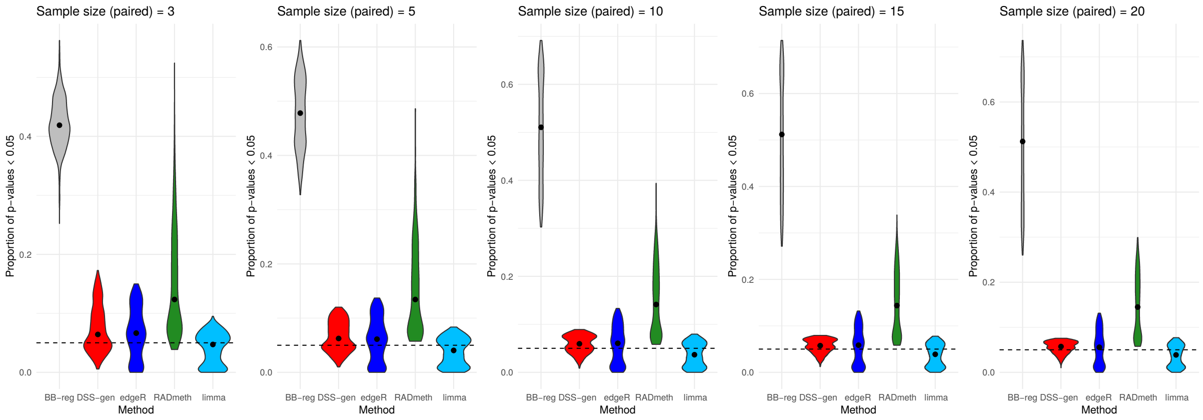

(c)

Supplementary Figure 2: Serial CPU time taken by the five candidate DML callers for all 3,360 simulations as a function of **(a)** coverage, **(b)** methylation shift, **(c)** τ and **(d)** covariate size.

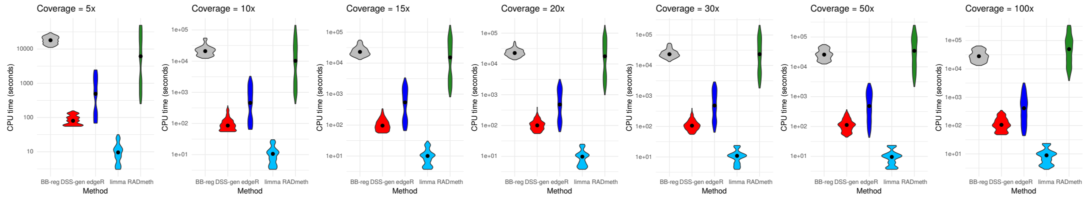

(a)

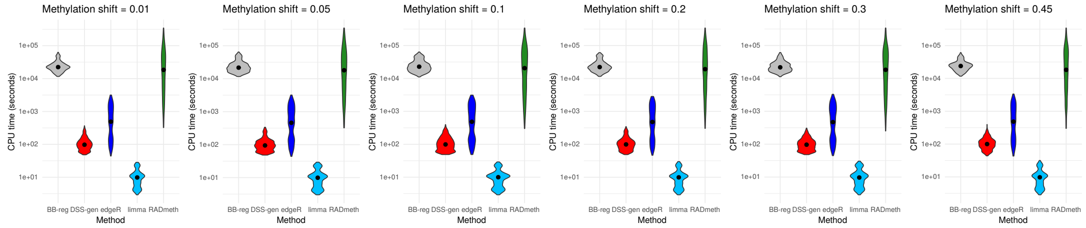

(b)

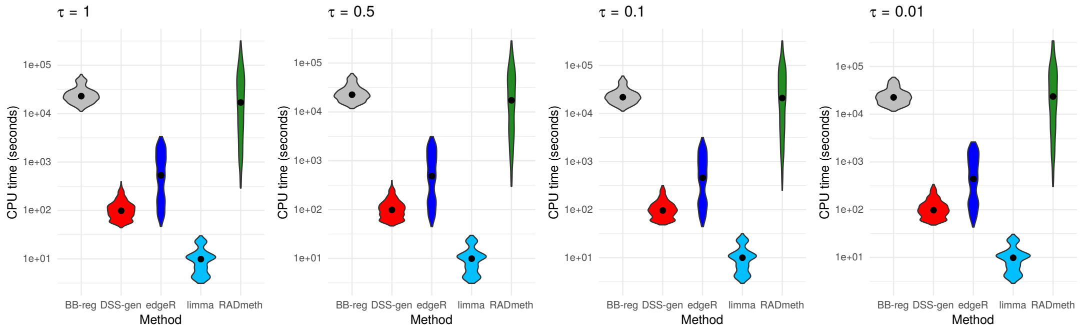

(c)

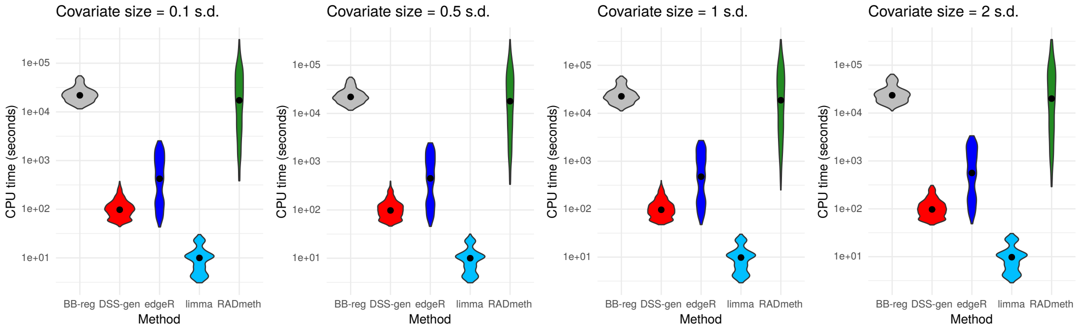

(d)

Supplementary Figure 3: **(a)** ROC showing predictive performance of four DMR callers for the non-depleted simulated dataset. **(b)** Data in Figure 5a grouped by method, rather than simulated coverage. **(c)** Predictive performance of DMRcate for various kernel sizes, on the same simulated dataset as for (a). **(d)** The same data as in (c) except grouped by simulated coverage. Colour legend for kernel sizes: 1000-black; 500-red; 200-green; 100-blue; 50-cyan; 20-magenta; 10-orange.

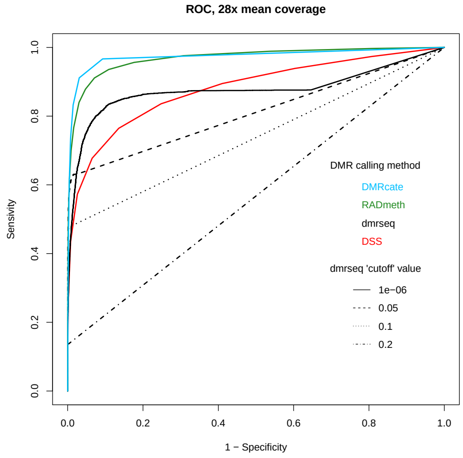

(a)

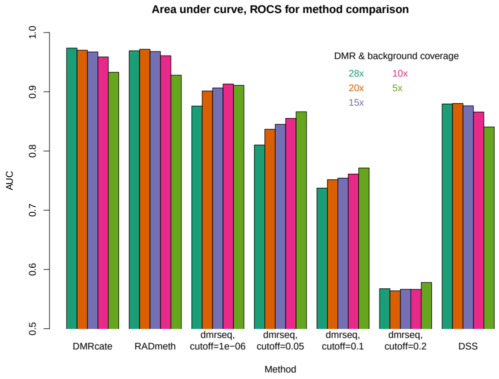

(b)

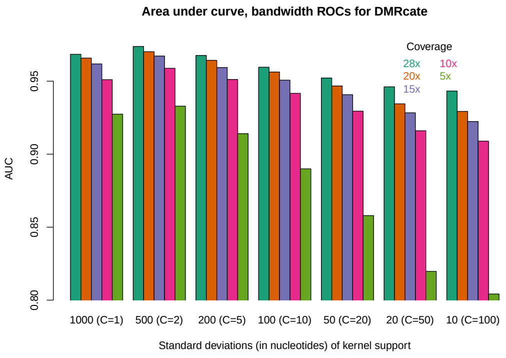

(c)

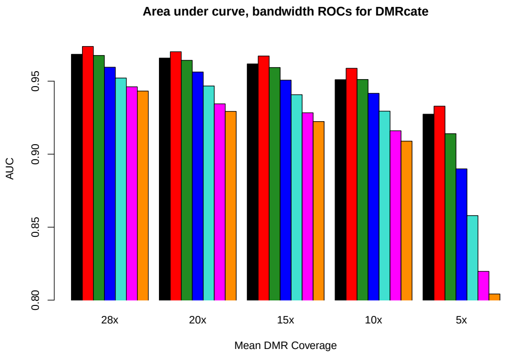

(d)
